# Truth Is Mighty, Etc.

**Published:** 1871-09

**Authors:** 


TRUTH IS MIGHTY AND WILL PREVAIL.—"The Profession of Georgia is in a most bitter and unfortunate quarrel. Can any one give, briefly, the facts for publication? So far as can be ascertained here, the Faculty of the Atlanta Medical College is generally, and justly, censured for its course."—*Richmond and Louisville Medical Journal.*

This does not look like "*peace.*" We ardently desire *peace* upon sound, correct, ethical principles among the Profession in this State. We deplore the necessity, which has forced the upholders and supporters of law in our State, to collide with those who have defide the law, and persist in overthrowing ethichal rule for the purpose of lowering the standard of Medical Education, and thereby degrading professional dignity. As long as this continues there can be no professional peace in Georgia. When peace *does*

come, we will be able to *proudly* point the Profession to the *time*, *place*, and the *terms*. Depend upon it—there will be no "shaking of hands" *until principle is established and the law vindicated.* Never will we, or the Profession of the State, compromise truth in order to render error respectable.

A full and faithful history of the controversy alluded to above, has been published by the Fulton County Medical Society, compiled from the records. A copy will be forward to any one desiring it, upon application, with postage stamp enclosed, to the Editors of The Companion.

